# Improvement and Validation of a Smart Road Traffic Noise Model Based on Vehicles Tracking Using Image Recognition: EAgLE 3.0

**DOI:** 10.3390/s25061750

**Published:** 2025-03-12

**Authors:** Claudio Guarnaccia, Ulysse Catherin, Aurora Mascolo, Domenico Rossi

**Affiliations:** 1 Department of Civil Engineering, University of Salerno, Via Giovanni Paolo II 132, I-84084 Fisciano, Italy; amascolo@unisa.it; 2 Laboratoire des Sciences du Numérique de Nantes, Ecole Centrale de Nantes, 1 Rue de la Noë, F-44300 Nantes, France

**Keywords:** video analysis, image, recognition, vehicle tracking, CNOSSOS-EU emission model, road traffic noise assessment, field measurement validation

## Abstract

Noise coming from road traffic represents a major contributor to the high levels of noise to which people are continuously exposed—especially in urban areas—throughout all of Europe. Since it represents a very detrimental pollutant, the assessment of such noise is an important procedure. Noise levels can be measured or simulated, and, in this second case, for the building of a valid model, a proper collection of input data cannot be left out of consideration. In this paper, the authors present the development of a methodology for the collection of the main inputs for a road traffic noise model, i.e., vehicle number, category, and speed, from a video recording of traffic on an Italian highway. Starting from a counting and recognition tool already available in the literature, a self-written Python routine based on image inference has been developed for the instantaneous detection of the position and speed of vehicles, together with the categorization of vehicles (light or heavy). The obtained data are coupled with the CNOSSOS-EU model to estimate the noise power level of a single vehicle and, ultimately, the noise impact of traffic on the selected road. The results indicate good performance from the proposed model, with a mean error of −1.0 dBA and a mean absolute error (MAE) of 3.6 dBA.

## 1. Introduction

Assessment of noise is currently a major concern for urban settlements. According to the European Environment Agency (EEA), a large and growing number of people are constantly exposed to harmful levels of noise (exceeding 55 dBA) [1]. The consequences of such exposure can be various, as reported in the literature: people exposed to continuous harmful levels of noise can experience irritability, deprivation of sleep, lack of concentration, transient or permanent hearing loss, and even blood pressure issues [2,3,4,5,6,7,8,9,10,11]. In [12], various symptoms related to hypertension are clearly correlated with high noise level exposure. As demonstrated in [13], moreover, even if indirectly, noise can generate stress that then promotes psychological symptoms and disorders, finally leading to brain and cardiovascular disturbances. Noise not only influences people’s regular life in urban contexts, but it may be extremely harmful to specific categories of inhabitants. Pupils attending school in particularly noisy areas, in fact, are seen to exhibit worse performance in their studies compared to students attending other institutes [14]. Elderly people are also particularly sensitive to relevant noise levels, as demonstrated in [15]. While it is clear in the literature that exposure to high levels of noise is dangerous for human health, it is also clear that the main contributor to this hazardous noise is recognized to be the transportation system [1]. In particular, noise coming from vehicles flowing on urban roads is responsible for several health consequences for inhabitants, although other transportation sources (railways and airports) [10] and noise coming from other human activities [16] are not negligible.

Even if limitations of noise levels are often compelled by law, the situation is still critical, and local governments, as well as national and supranational ones, are showing more and more interest in developing a proper road traffic noise evaluation [17]. Other studies relating the exposure to noise, and therefore the consequent rate of complains, to the socio-economic factors of people, found that cities/regions with a higher proportion of young and single residents tend to have more noise complaints, as do cities/regions with diverse ethnicities and religions [18].

Due to this situation, the European Union has solicited important and urgent mitigation actions to be implemented to reduce noise levels in European cities and therefore diminish their effects on human health. The EU Environmental Noise Directive establishes the ways such mitigation actions have to be realized. In the literature, some examples of plans are retrievable: in [19], a noise mitigation action is implemented using road degradation and the optimization of arterial roads; in [20], the authors propose a traffic noise prediction method to find the upper limit of network noise emission based on design elements. A traffic flow detection using camera images and machine learning methods is also proposed in [21] as a strategy for effective noise reduction in a given urban context. A comprehensive review of innovative approaches for noise management and mitigation is reported in [22].

In this framework, for the assessment of noise coming from road traffic, two ways are technically possible: one is the direct measurement of noise levels, and the other is their simulation. Direct noise measurement is generally the preferred solution, but many issues can make it difficult or even impossible. Direct noise measurements require dedicated instrumentation, such as sound level meters, which are generally expensive, fragile, demand constant maintenance, and need to be used by qualified personnel. Before and after their use, they also necessitate a proper calibration, which contributes to making their usage not simple and sometimes even difficult. Finally, the conformation of the urban roads under analysis may not permit a proper arrangement of the instrumentation, making the measurements de facto impossible [23,24].

Simulating road traffic noise, on the other hand, has its advantages. First, it takes away the majority of the aforementioned problems; then, it is a generally flexible approach, which can also be used to discover the road traffic noise impact on an infrastructure under construction. Models are often preferred to a direct measurement, and many of them have been built and validated over the years, starting from the early 1950s; these models are known as road traffic noise models (RTNMs). By using different formulas, these RTNMs have been built to assess the sound power level of flowing lines of vehicles (or of a single vehicle), generally assuming free flow conditions, starting from the following road traffic inputs: the density of flowing vehicles, percentage of heavy vehicles, and their speed. RTNMs have been developed by different countries, and therefore they have been used as national models. They include the CoRTN model [25], the SonRoad model [26], the NMPB model [27], the RLS90 model [28], the Harmonoise model [29], and also the model developed by Quartieri et al. [30]. Recently, the European Union member states joined their efforts to produce a more comprehensive method for the assessment of the impact of road traffic noise on sensible receivers (also using mapping), resulting in the implementation of the CNOSSOS-EU model [31]. Such a model, moreover, aims to unify the modeling of noise in all European countries that are obliged to use this model for strategic noise mapping. A comprehensive description of these models, their peculiarities, and the history of their generation can be found in [32].

Important precautions are required in order to build a proper model, and the first and main attention to be paid is a proper collection of input data. RTNMs, in fact, take vehicular data as input and process them, giving as output a final noise level—usually an equivalent noise level. Typical input parameters used are the vehicle number and speed and the percentage of heavy vehicles over the whole amount. In case such input data are not properly collected, the whole process of calibration of the model will conclude with an inefficient model. Input data can be measured, collected, or even simulated, such as in [33,34].

A proper process of collecting data is then essential for a good modeling result. Data collection campaigns, anyway, are not the only way possible to retrieve useful data to feed models. In recent times, in fact, an increasing number of monitoring stations have been developed, especially in urban areas. Such stations, sometimes compelled by law, are able to collect a large amount of data, often in a continuous way, furnishing then an available input for any model. Such stations can be strictly acoustic, can combine meteorological and acoustic collections [35], or can be even more complex. Another type of station involves video recording, by which it is possible to obtain multiple types of information regarding the road traffic data like the number and type of flowing vehicles: this kind of data collection is used in the presented research. Here, in fact, the authors present a new method for gaining road traffic input data like the speed and position of a single vehicle from a video capture of the traffic on an Italian highway, A2 “Autostrada del Mediterraneo”. The presented approach has its groundwork in an already published procedure—defined as EAgLE, Equivalent Acoustic Level Estimator [36,37]—which is here resumed and innovated. In the original work, out of several video recordings of flowing vehicles, typology and speeds of each of them were retrieved and then, consequently, used to estimate their contribution in terms of acoustic energy at the receiver. Procedures were applied both on a national road in Portugal and on a portion of an Italian highway. The ground idea of using video tracking to retrieve road traffic information is not new, since several examples can be found in the literature. In [38], as an example, the authors report an interesting tracking procedure for car recognition during the day and night by distinguishing dark and bright areas on the video. In [39], a real-time highway surveillance system is described, working to collect speed information of vehicles. A real-time vision system for automatic traffic monitoring based on a network of autonomous tracking units is presented in [40]. In [41], the authors implement a tool including low-cost cameras and a vehicle recognition and counting method to provide updated data over time for noise mapping.

In the described methodology, recurring to a more advanced video capturing analysis, the trajectory and the speed of vehicles are retrieved and a contemporary classification is performed to differentiate the noise contribution of light and heavy vehicles. The presented methodology is based on two already published works [36,37], where for the first time authors built a road traffic noise model starting from a recorded video, counting vehicles crossing an intrusion line using an image background subtraction technique. Starting from the original idea, in the presented paper, the authors ameliorated the vehicle automatic recognition tool in order to have a more accurate detection of the flowing vehicles. In particular, the most important novelty is related to the implementation of the detection of the trajectories of each single vehicle, which permits the estimation of the noise contribution at the receiver as a function of time. Such a finer noise analysis that has been conducted provides a time-dependent assessment of the overall noise contribution of all the vehicles flowing, describing the pressure level progress at a single-second time step and obtaining a “time history” of the noise impact on the investigated receiver.

The procedure is divided into four main steps: an inference step to obtain the vehicles’ positions, a geometric step in which the real vehicles’ positions are retrieved, a data treatment during which the obtained results are filtered, organized, and augmented, and a final step of noise analysis to compute the continuous equivalent sound level. When comparing with measured data, the obtained simulated results indicate a good accuracy of the model, opening to the possibility of using the presented video procedure for an automatic collection of input data to feed any road traffic noise model, obtaining proper and valid output simulations of the related noise into a given environment.

## 2. Materials and Methods

### 2.1. Case Study Description

**Figure 1:**
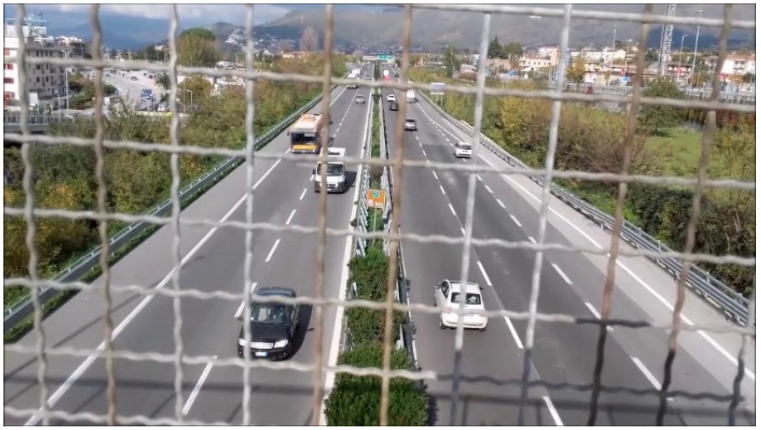
Point of view of the camera recording the traffic flow from the bridge on the highway, which is the same as in [36].

The location selected as a case study in this paper is the same as in [36]. Specifically, an early implementation of EAgLE has been carried out on an Italian site highway—the A2 “Autostrada del Mediterraneo”. A bridge overcoming the chosen highway in the city of Baronissi was selected as the location for video recording and measurement with a sound level meter, as shown in Figure 1. This particular stretch of the highway includes two lanes in each direction, plus an entering lane towards the north that comes from a gas station. The equipment was set up in a completely safe manner on the sidewalk of the bridge, where both audio and video recordings were performed. For sound recording, the authors used a class 1 sound level meter Fusion by 01dB Acoem^®^, Limonest, France, equipped with a cover to protect it from possible abrupt wind peaks. The sound level meter was properly calibrated before usage with a certified standard signal of 94.1 dB at 1000 Hz. The measurement was performed supervised by operators, who, in addition to handling the instruments, performed the manual counting of the vehicles. As for video recording, the authors used a camera embedded in a mobile phone. Measurements were performed around lunchtime on Friday, 17 November 2017, and they were organized into two measurements of 15 min. Instantaneous sound pressure levels A-weighted with the “Fast” time constant (*L_p,A,F_*), A-weighted continuous equivalent levels (*L_eq,A_*), percentile levels, acoustic spectrum in third octaves, and other acoustic parameters were measured, in parallel with the video recording of passing-by vehicles. During the measurement session, no unusual events were detected (anomalous transits, honking, and airplanes, among others). Moreover, the vehicles’ flow on the bridge was negligible, as well as the flow entering the highway from the gas station. Due to this and also to the position of the sound level meter, the authors could neglect the vehicle transit behind the measuring position. The sound level meter was mounted on a tripod support to assure stability. A thorough description of the field measurement campaign can be found in [36]. The environmental temperature during the measurement was between 11 °C and 14 °C, with a wind speed always below the standard limit of acoustic measurement validation. Anyway, a windshield was mounted on the microphone to reduce the impact of wind on noise levels. The traffic flow runs in almost steady conditions, with an average number of vehicles of 1091 in 15 min. The percentage of heavy vehicles was about 15% in both measurements.

### 2.2. Software and Resources

The majority of the procedure was implemented using libraries under Python 3.13.2, including some that are currently under development. The vehicles’ recognition communicates with Roboflow’s API thanks to Roboflow’s libraries supervision 0.19.22 and inference 0.9.22. Roboflow is a set of computer vision datasets that can integrate with many other platforms, used to detect and track objects on video. Roboflow is widely used both in the literature and in industries for this scope [42,43], and it is very easy to implement with Python code. Models implemented by using Roboflow datasets (such as YOLO, for example) are widely used in the literature for the identification and tracking of objects [44,45,46].

Other standard libraries were used for the data analysis, such as pandas 2.2.1, cv2 (opencv-python) 4.9.0.80, NumPy 1.26.3, matplotlib 3.8.3, and bottleneck 1.3.8. The whole procedure was implemented on a personal computer with the following specifications: 3.50 GHz CPU with 16 GB of RAM installed, 64-bit.

## 3. Model Implementation

**Figure 2:**
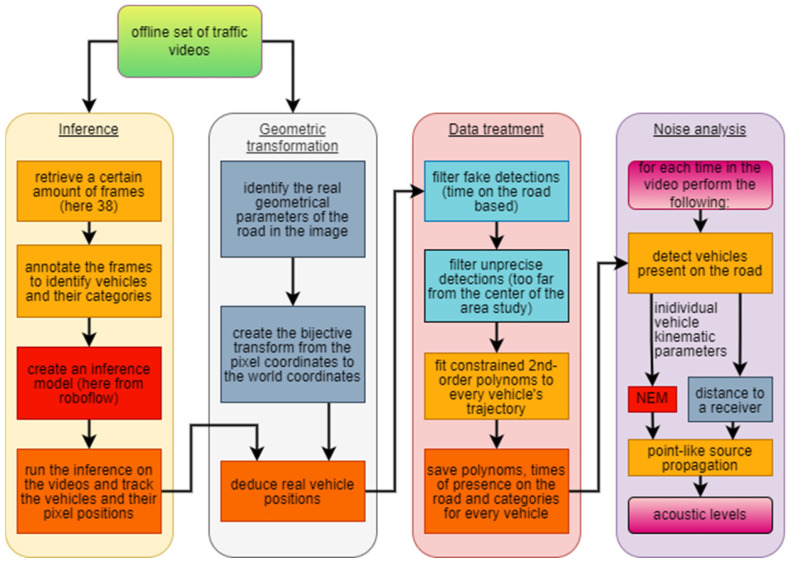
Flow chart of the proposed methodology. The four parts of the analysis are sequentially reported, with the connection between each step.

The model implementation is described in this section and resumed as a flow chart in Figure 2.

### 3.1. Inference

The most relevant novelty of the presented approach concerning the previous models is the enhancement of the accuracy of vehicle detection and tracking through an image inference process. The previous models, in fact, presented good accuracy on sample videos, as reported in [36], but also highlighted some limitations related to the presence of the metallic grid in the image, which hid partially or totally vehicles on some frames, and the focus of the camera that sometimes automatically lost the focus on the road.

**Figure 3:**
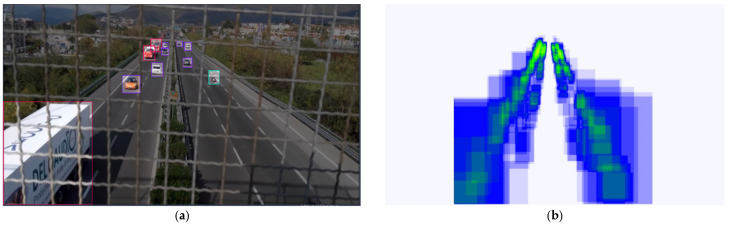
(**a**) Creation of the inference model; (**b**) every annotation position: a single detection box is light blue, and the more they are, the greener it becomes.

A subset of frames coming only from 2 video samples out of 10 were selected to create an inference model. Please note that the inference model was applied frame by frame and not on the whole video, mainly for reason of time and computational resources required. This has been performed to test the real validity of the model on the other samples of video that were not selected for the model training, to avoid overfitting issues. The Roboflow online tool for creating an inference model was used. A total of 38 images were manually annotated and used for the model, as shown in Figure 3a, while the annotation’s positions in the frames are displayed in Figure 3b.

The resulting annotations per category of the vehicle are detailed in Table 1.

The authors observed that the “Heavy vehicles” category was slightly under-represented to give a precise inference, and the “Two-wheels” category was slightly under-represented. The images dataset was then augmented to 98 images by flipping the images horizontally, changing the brightness by ±15%, and adding image noise up to 0.1% of pixels. For the model training, frames were separated as shown in Table 2.

**Figure 4:**
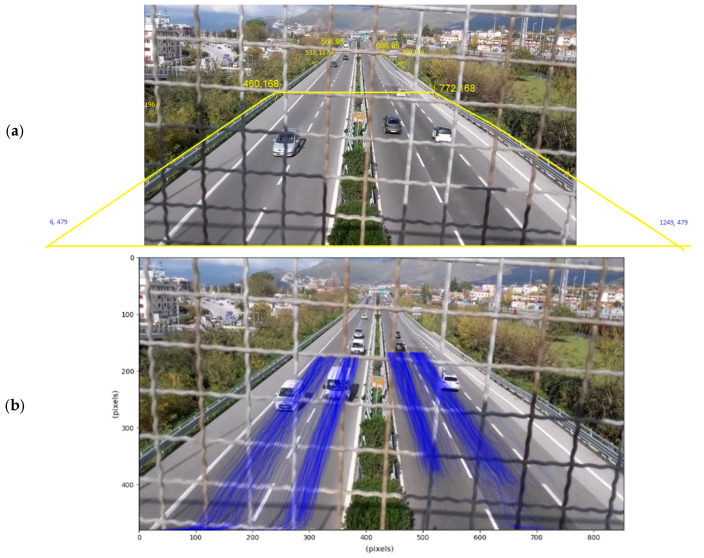
(**a**) Pixel coordinates of the sensitive area; (**b**) vehicle traces in pixel coordinates.

The model training ran for approximately half an hour, giving successful results on its frame tests. Various error metrics were evaluated to establish the running time, with a procedure directly embedded in the Roboflow algorithm. In detail, Box Loss, Class Loss, and Object Loss were evaluated. All of them fall down to values below 1.3 after 200 epochs. Then, associated with the modern powerful tracker Bytetrack [47,48], the model inferred every frame of every video sample. It retrieved each vehicle category, unique identifier, pixel position, and time. The sensitive area pixels and the vehicles’ trajectory detections on the video are indicated in Figure 4a,b, respectively. An example of the video analysis is provided in the Supplementary Material.

Such vehicle tracking represents a significant novelty compared to the methodology adopted in [36,37], where trajectories were not available, and counting was performed at a single point.

### 3.2. Geometric Transformation

This part does not require the model of inference and follows a standard procedure from Roboflow [49,50]. The objective is to accurately convert pixel positions from the video to real-world positions. It is a very common problem that, for instance, has been faced in [51]. From the image plane (*O*_1_*, x, y*) to the road plane (*O*_2_*, X, Y*), both in homogeneous coordinates, exists a homography matrix $H\;\in \;R^{3\times 3}\;$, where

(1) $$\left(\begin{array}{c} X\\ Y\\ 1 \end{array}\right)=H\times \left(\begin{array}{c} x\\ y\\ 1 \end{array}\right)$$

**Figure 5:**
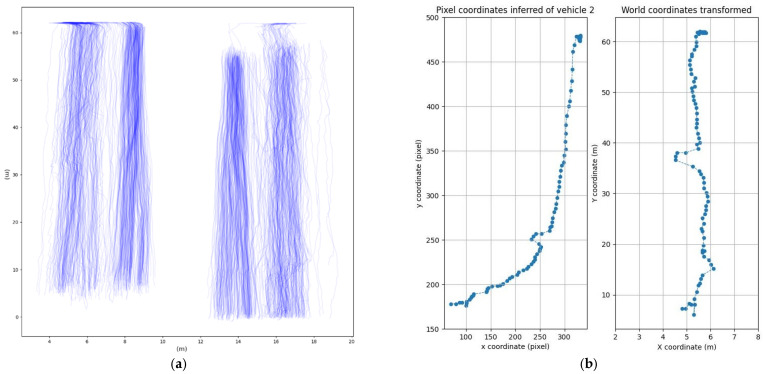
(**a**) Vehicle traces in world coordinates; (**b**) homography detail for a single vehicle.

This homography matrix is found using the cv2 package. This matrix can be automatically found with four noncolinear points, giving their world coordinates and pixel coordinates. The world coordinates were measured with the aid of satellite images and Google maps^®^. The real vehicle positions depicted in Figure 5a were then deduced from their pixel detections from Figure 4. An example of this transformation for a single vehicle is described in Figure 5b. All the trajectories’ points are not necessarily identified in subsequent frames of the video: sometimes the model cannot find the vehicle for a few moments because it is hidden by the grid or by another vehicle, but then the tracking finds the vehicle again. As a result, fake detections and some instability in the trajectories are present. The handling of these issues, which may have caused errors in the vehicle trajectory detection and the speed estimation, is faced in the next subsections.

### 3.3. Data Treatment

**Figure 6:**
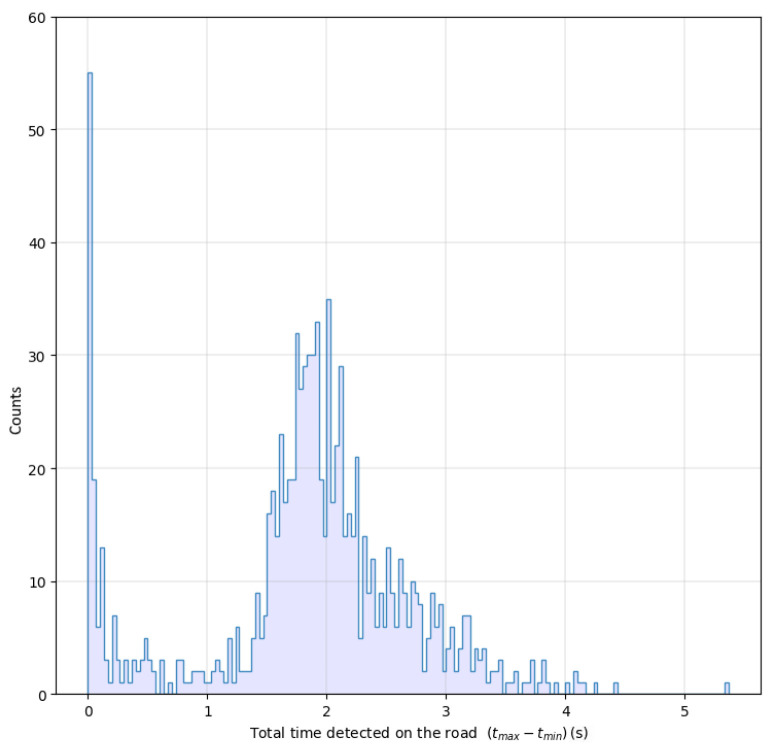
Distribution of the total time of vehicles on screen.

As mentioned above, the resulting positions for each vehicle are not stable, and at first, there are fake detections. The current time on screen detected for each vehicle has a non-normal distribution and presents a relevant peak at very low values, as shown in Figure 6. These vehicles, spending very little time in the detection area, can surely be labeled as fake recognitions and cause the problems described in the previous subsection.

**Figure 7:**
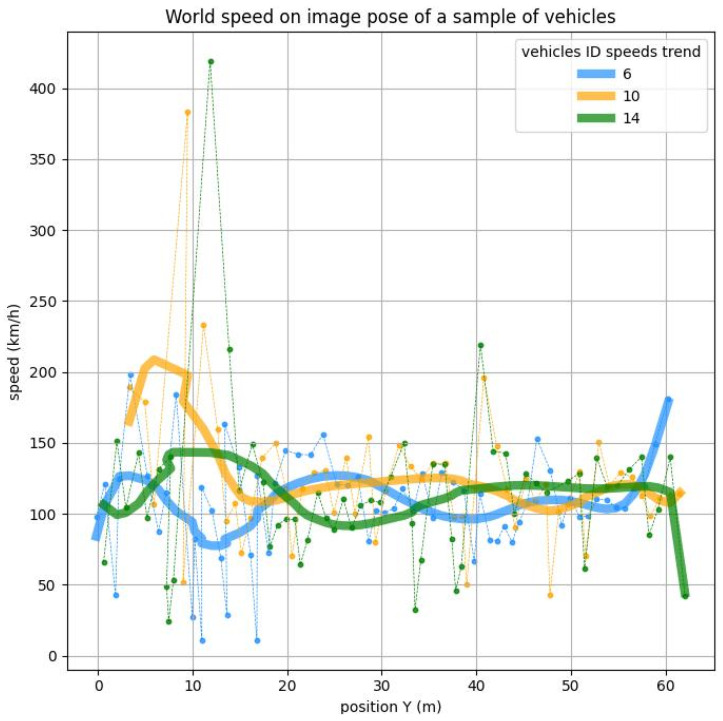
Speed over the position of a sample vehicle.

For these reasons, the authors chose to remove every vehicle that spends less than 1.2 s in the detection area. This threshold was chosen considering that the analysis will be performed over 40 m (see comment on boundary fake detection below). To cover this range in 1.2 s, the speed must be 120 km/h. Considering that the speed limit in this highway segment is 80 km/h, for both light and heavy vehicles, this choice also includes potential vehicles running faster than allowed. Another problem is that position estimation is imprecise when vehicles come in and come out of the inference window. This is because the homography’s precision diverges at the edges of the homography plan, and partially recognized vehicles at the edge of the inference window artificially shift the vehicle’s positions. The instantaneous speed results in large errors in these areas, as shown in Figure 7.

To overcome this problem, data from the boundary positions were removed to keep only positions included in the range 15 m < Y < 55 m. Since the objective is to obtain the kinematic parameters of every vehicle, in particular position and speed, as inputs for a road traffic noise emission model, the authors exploited some different methods of smoothing or filtering to apply to the dataset.

The first one is a second-order polynomial fitting of the position. The authors constrained the second-order factor of the polynomial, which represents the acceleration of the vehicle, to a maximum value that reflects a maximum acceleration of 6.9 m/s^2^. The second method is a third-order polynomial fitting, without any restriction on the maximum value of acceleration that would become heavier to handle. The third method is a Savitzky–Golay algorithm [52], used in signal processing. This method fits a polynomial of the desired order to a restricted number of adjacent values of the signal to extract the smoothed value at the center of the adjacent value window. This window slides repetitively to each value of the signal. This method allows the direct derivation of the signal while smoothing it to any order because the polynomials extracted can be analytically derived. The final method is a Butterworth filter, a linear filter designed to be as constant as possible in its passband [53]. It was applied to the instantaneous speed directly because it is supposed to be used on averagely constant signals.

**Figure 8:**
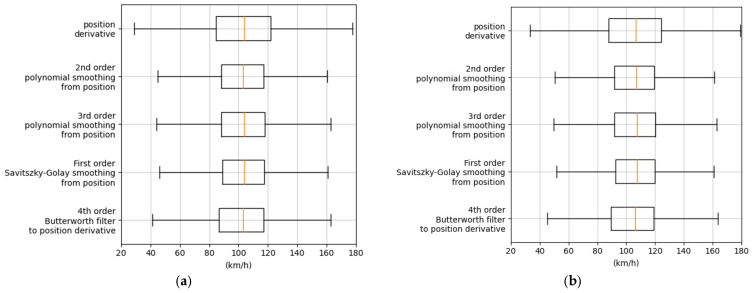

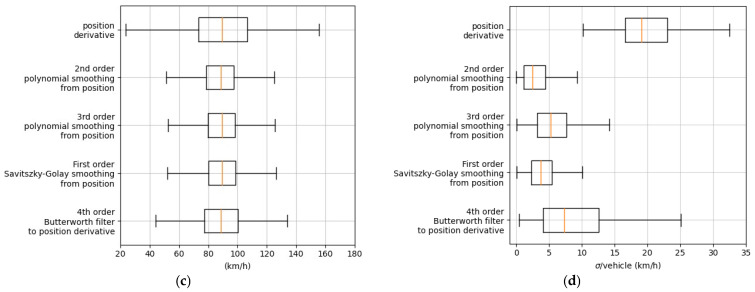
(**a**) Comparative method speed boxplot; (**b**) boxplot of the distribution of light vehicles’ speed; (**c**) boxplot of the distribution of heavy vehicles’ speed; (**d**) standard deviation speed per vehicle. Orange lines in the boxplots are the median values of the distributions.

A comparison of the outcomes of these methods is shown in Figure 8a and also plotted separately for light vehicles in Figure 8b and for heavy vehicles in Figure 8c. The authors observed that heavy vehicle speeds were more precisely estimated; this is probably due to easier detection of a bigger object on the video and to the fact that heavy vehicle speeds are on average lower and more controlled due to the professional behavior of their drivers. Due to the characteristics of the analyzed road, moreover, the authors expected to have a narrow distribution of speeds. In the recorded portion of the highway, in fact, a free flow of vehicles was observed. For this reason, the values obtained by the described methodologies were expected to be normally distributed and reasonably peaked, since no abrupt speed changes should occur. In Figure 8d, the standard deviation of the speed of each single vehicle is shown in relation to the smoothing methodology applied.

Looking at the mean and standard deviations of each fitting procedure reported in Table 3, it is possible to notice that the main differences are related to the spread of the distributions rather than to their mean values. The mean values of light and heavy vehicles, in fact, are coherent for all of the smoothing processes, while the standard deviations are different when considering the derivative of the position or the other smoothing processes. The parameter changing the most is the sigma over the speed of vehicles, which exhibits a quite large variation between median values over the different fitting procedures. Since the speed during each transit in the sensitive area is expected to have small variations due to the steady free flow, the second-order polynomial smoothing was selected because it exhibits the lowest (3.22 km/h) mean value and the second-lowest standard deviation value (3.53 km/h).

The above-described procedure allows the detection of vehicles in a window of 40 m, i.e., the length of the sensitive area, but the noise they emit when they are out of this window cannot be neglected. That is why the position and speeds of each vehicle were artificially deduced out of the window of detection for a total length distance on which the vehicle is considered to have a non-negligible impact on noise of roughly 600 m. This value was selected considering an ambient noise of 50 dBA and the minimum distance to which a car at high speed would emit the same noise level and thus become relevant to the sound environment.

### 3.4. Noise Estimation

The speed *v* and category m of vehicles are the only parameters required to feed the single-vehicle noise emission model (NEM) of CNOSSOS-EU. The single-vehicle sound power level *L_W,i,m_* per octave band is computed as follows:

(2) $${L_{W}}_{,i,m}\left(v\right)=10\times \log_{10}\left(10^{\frac{{L_{WR}}_{,i,m}\left(v\right)}{10}}+10^{\frac{{L_{WP}}_{,i,m}\left(v\right)}{10}}\right)$$

where *L_WR,i,m_* and *L_WP,i,m_* are the noise power levels for rolling and propulsion contributions, respectively. According to the CNOSSOS-EU model, rolling describes the noise contribution coming from tire–road interactions and aerodynamics, while propulsion describes noise contribution coming from the engine, transmission gears, exhaust system, and other mechanical parts. Their formulas are given in the Equations (3) and (4), with *A_R,i,m_, B_R,i,m_, A_P,i,m_, B_P,i,m_* being coefficients of the model for rolling (the first two) and for propulsion (the last two), respectively. Please note that the original coefficients given in [31] have been subsequently amended with new ones that were found in [54] and made official in [55]. For these formulas, the authors used such new coefficients.

(3) $${L_{WR}}_{,i,m}\left(v\right)={A_{R}}_{,i,m}+{B_{R}}_{,i,m}\times {log}_{10}\left(\frac{v}{v_{ref}}\right)$$

(4) $${L_{WP}}_{,i,m}\left(v\right)={A_{P}}_{,i,m}+{B_{P}}_{,i,m}\times \left(\frac{v-v_{ref}}{v_{ref}}\right)$$

A virtual receiver was chosen at 10 m above the road, at the center of the study area. This location was specified because it is the position in which the sound level meter was installed during the measurements and allows the validation of the output of the model over the field-collected data.

**Figure 9:**
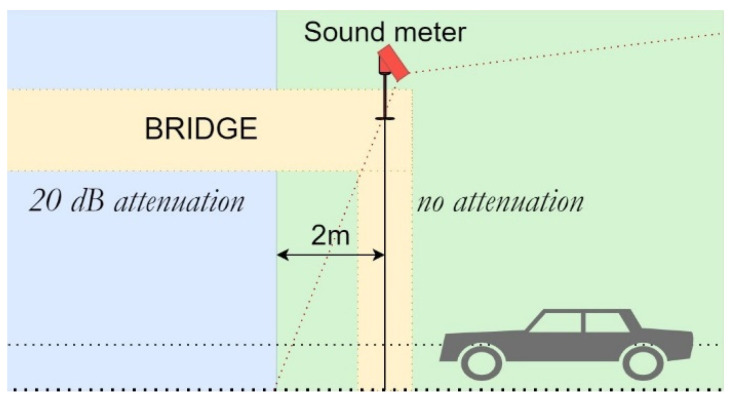
Schematic representation of the bridge attenuation.

To consider the real attenuation due to the 15 m wide bridge the sound meter was on, *L_W_* values of every vehicle below 2 m after the sound level meter were attenuated at a fixed value of 20 dBA, as illustrated in Figure 9. This is a strong, however realistic assumption.

To compute the final *L_p_* overtime at the receiver, the model starts by calculating the single-vehicle sound pressure level at the receiver, *L_p,j_*, considering *L_W,j_(t)* as the noise power level of vehicle *j* at time *t* and *d_j_(t)* its distance to the receiver, using the point-like source formula of a spherical emission, with absorbing ground:

(5) $$L_{p,j}\left(t\right)=L_{w,j}-20\log_{10}\left(d_{j}\right)-10\log_{10}\left(4\pi \right)$$

The overall noise level through time is then obtained with the logarithmic sum over the vehicle present in the detection area at time *t*:

(6) $$L_{p}\left(t\right)=10\log_{10}\left(\sum_{j}10^{\frac{L_{p,j}\left(t\right)}{10}}\right)$$

To compare these noise values through extended periods, the continuous equivalent sound level *L_eq,A_* quantity is used as expressed in Equation (7).

(7) $$L_{eq}=10\log_{10}\left(\frac{1}{T}\int_{t\in T}10^{\frac{L_{p}\left(t\right)}{10}}dt\right)$$

At this stage, the background noise level is not considered since the continuous traffic flow allows us to neglect it.

## 4. Results and Discussions

From each of the two 15-min recordings of the camera on the Baronissi highway, 5 video samples of 1 min were extracted, resulting in 10 samples of 1 min each. Those video samples were then analyzed to manually count vehicles and compare the results with the automatic counting performed by EAgLE. The results are given in Table 4. The video samples were labeled according to the number of the original video (first digit) and to the partition made by the authors (digit after the point).

**Figure 10:**
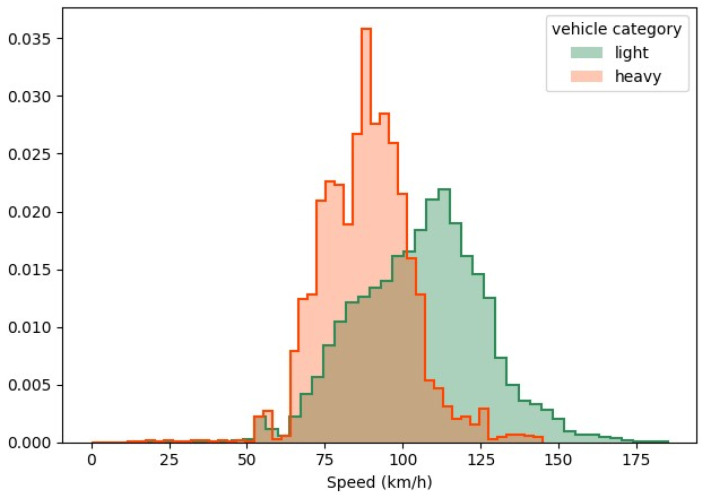
Distributions of the estimated vehicles’ speed. Distributions were normalized to 1.

As a conclusion, the global vehicle detection precision is about 98%. The world positions deduced from the inference were smoothed to estimate speeds, which were then augmented to a bigger portion of the road, as described in Section 3. The final estimated speed distributions are displayed in Figure 10 for both light and heavy vehicles, in which it can be seen that the two peaks of the distributions are related to the speed limits of each category.

**Figure 11:**
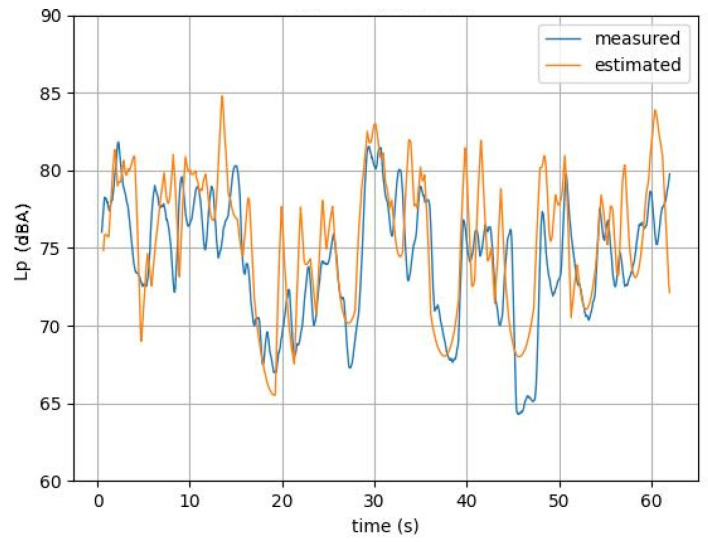
Measured and estimated *L_p_* moving average curves over time of the 2nd video sample.

The deducted *L_p_* values over time were then plotted for the ten video samples as a function of time. Figure 11 shows one of them. A moving average was computed on the data to remove ripples in the simulated time history signal and to compare with the measured pressure levels.

The measured continuous equivalent sound level was compared to the estimated one on the selected 10 slots of 1 min, obtaining the following result:

$$\left\{\begin{array}{c} L_{A,eq,meas}\\ L_{A,eq,est}\;\\ {∆L}_{A,eq} \end{array}\begin{array}{c} =\\ =\\ = \end{array}\begin{array}{c} 76.3\\ 78.0\\ -1.7 \end{array}\begin{array}{c} \;dBA\\ \;dBA\\ \;dBA \end{array}\right.$$

**Figure 12:**
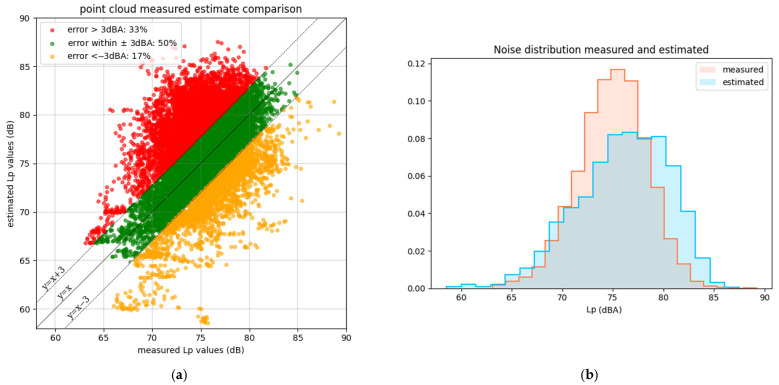
(**a**) Scatterplot of measured vs. estimated values of noise; (**b**) distributions of *L_p_* through time.

Additionally, the scatterplot of the estimated versus measured noise pressure levels in each time step is displayed in Figure 12a. Figure 12b shows the *L_p_* distributions.

The different error metrics were calculated on the pressure level distribution and are reported in Table 5.

### 4.1. Cross-Model Comparison

**Figure 13:**
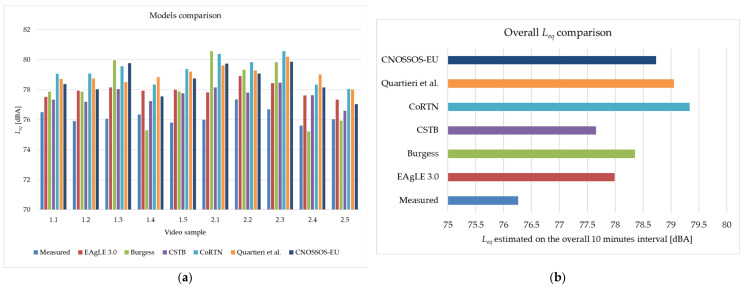
(**a**) Barplot of measured and simulated equivalent levels in each video sample; (**b**) comparison between *L_eq_* measured and estimated by the selected models on the overall 10 min interval. The models used are Burgess [56], CSTB [57], CoRTN [25], Quartieri et al. [30], and CNOSSOS-EU with updated coefficients [55].

A cross-model comparison was performed to test the performances of the EAgLE 3.0 model with respect to standard and well-known road traffic noise models, such as Burgess [56], CSTB [57], CoRTN [25], Quartieri et al. [30], and CNOSSOS-EU with updated coefficients [55]. The test was performed on each 1.1 video sample and the overall 10-min interval. Continuous equivalent levels in each 1 min interval and in the overall time range are plotted in Figure 13a,b, respectively. Mean error (calculated as the mean of the difference between the measured and simulated *L_eq_*) and sample standard deviation of the errors were calculated per each model and resumed in Table 6.

It can be noticed that a general overestimation is present. All the models tend to provide noise levels higher than the measured ones, probably due to the free-field assumption of the models and to the presence of the bridge that screens part of the transit track. In addition, the models were developed to work in a lateral position, rather than from the top, as in our case study. This condition may introduce an additional systematic error. Anyway, the overestimations shown by the mean errors of the literature models are lower than 3 dBA, probably due to the free flow and highway conditions, which are close to the reference settings by which the models were calibrated.

Looking at the EAgLE model, it can be noticed that it performs well in basically all the video samples, showing a good mean error and the lowest standard deviation. Moreover, it is interesting to compare EAgLE with CNOSSOS-EU. The former model, in fact, shares the same noise emission model (NEM) of CNOSSOS-EU but differs from the latter in the aggregation of single vehicles to the traffic flow source and propagation. It can be noticed that the EAgLE outputs are closer to the measurements, demonstrating that a microscopic and dynamic approach can improve the estimation of road traffic noise levels. Further investigations, aimed at deepening the tuning of the hyperparameters and the parameters, could enhance the proposed approach and possibly outperform the existing techniques.

### 4.2. Discussion and Limitations

This contribution combines different fields, starting from image analysis, machine learning, and acoustics, to achieve a microscopic and dynamic road traffic noise model. The error metrics reported in Table 4 can be considered fully compliant with the expectations of a model that needs to provide accurate predictions of urban and non-urban sound levels. According to ISO 9613-2/1996 [58], in fact, the typical standard error in outdoor sound propagation models should be approximately ±3 dBA. The European CNOSSOS-EU method [31], used for strategic noise mapping, considers acceptable deviations of ±2 dBA between measurements and predictions. Some national models (such as the French NMPB, the British CRTN, or the German RLS-90) specify that the difference between measurements and calculations should generally remain within ±2–3 dBA. For these reasons, the performances of the presented model are aligned with the literature expectations, making it a good choice for applications in which it is not possible to manually count the vehicles and measure the speed or when a dynamic and real-time assessment is needed.

On the other hand, inaccuracies were encountered, some of them related to the data collection the authors worked on. At the time of the measurement, the background noise could not be estimated because of constant traffic flow from the highway. This led to the impossibility of adding to the simulation of the background noise, considering the ambient contribution to the overall sound levels. The model, in fact, estimates only road traffic noise. Anyway, the validation was still good because of the negligibility of background noise concerning the pressure levels produced by the highway traffic flow in the considered time range.

The video recordings were made with non-professional equipment, without a stabilizer, and thus are degraded because of little movements of the camera due to the wind. In addition, the automatic camera focus sometimes moved from the road to the metallic grid in the foreground. This caused errors in the tracking of the vehicles, resulting in non-stable trajectories and, consequently, in strong variations in the speed estimation, as shown in Figure 7. These issues have been handled by filtering the best video segments and selecting for the model validation a total time of 10 min of video over 30. In addition, a fitting procedure on the estimated position was performed, as documented in Figure 8 and Table 3, assuming that the free and steady flow conditions result in a quite stable speed of the vehicle, without abrupt variations.

The inference model is surely ameliorable, and its improvement can be pursued through different actions. At first, training the model on a larger database could make the calibration more stable and secure. The usage of a limited number of frames, in fact, resulted in an under-representation of some categories, such as, for instance, two-wheel vehicles. A finer categorization of the detected vehicles would better match the real flow, including other categories than light and heavy vehicles. The used noise emission model, in fact, considers four categories (light, medium–heavy, heavy, and two-wheel vehicles) and thus can provide a finer assessment of the emission of the traffic flow. The used homography technique could be tuned way more finely, involving more advanced statistical methods [51] (that will require, anyway, a significant increase in the required computational effort).

Anyway, despite such possible improvements, the model provided a good level of accuracy, obtaining good results both in terms of video recognition performance and in the consequent noise emission prediction.

## 5. Conclusions

In this contribution, an improvement of an already published procedure for road traffic noise assessment based on video recording was presented. The method presented here is based on a Python script and is composed of two steps: the inference step and the geometric transformation step. In the first one, the recorded video is treated to obtain an automatic count of all the vehicles flowing, their type, and the final trajectory. In the second step, the position and speed of vehicles in real metrics are obtained. Following this double implementation, the noise estimation procedure was obtained by using the CNOSSOS-EU framework. Interesting results were found, since for the automatic vehicle count a maximum of 5 vehicle misalignments (over a total of 75) was found, indicating a low error on the first step of the procedure. The overall precision was 98%. Consequently, the second step of the noise simulation similarly ended in a very accurate model, having a mean error of 1.0 dBA and an MAE of 3.6 dBA.

The present approach, therefore, correctly works for road traffic noise assessment, joining in a single procedure both the obtaining of the input for road traffic noise calculation and their utilization for the actual assessment of the noise level. Due to its direct and straightforward way of recognizing and tracking vehicles, such a procedure could be easily implemented wherever a video camera can be installed. Two interesting applications could be on the highway (since cameras are—or can be—well distributed all along the road networks) but also in urban environments, wherever a video surveillance device is present. In any application, anyway, the model would require proper calibration, which is different according to the site.

The main limitations of this work lie in the error borders for vehicle detection and characterization from the recorded video since, up to now, some misclassifications are still present. Since the proposed model of vehicle recognition was built to be easily coupled with multiple noise emission models, future steps of this work will surely be devoted to the implementation of more models rather than the CNOSSOS-EU, which is actually the only one presented, so as to obtain a more complete tool for road traffic noise procedures. Moreover, in the next implementations of this procedure, other issues will be faced by adding more image frames in the calibration of the inference model. This will help in considering different vehicle categories that might have different noise emission curves to achieve a finer estimation of the source power levels and the overall sound levels. Finally, implementation of the described procedure in cities is planned, where low speed, pulsed flow, and congestion are more frequent than on highways. Such an application will surely require a finer tuning of the tracking algorithm but will permit an easy and valid estimation of road traffic noise in an urban scenario, where many more people are subjected to its detrimental effects.

## Figures and Tables

**Table 1:** Annotation distributions.

Category of Vehicle	Number of Annotations
Cars	259
Heavy vehicles	54
Two wheels	1

**Table 2:** Learning, validating, and testing number of frames.

Category of Vehicle	Number of Frames
Learning	90
Validating	2
Testing	6

**Table 3:** Mean and standard deviation of the speed for all vehicles, for light vehicles, and for heavy vehicles for each smoothing process. The last raw reports the mean and the standard deviation for the sigma per vehicle for each smoothing process.

	Position Derivative	2nd-Order Polynomial Smoothing	3rd-Order Polynomial Smoothing	Savitsky–Golay	Butterworth Filter
All vehicles					
mean	103.36	103.00	103.73	103.59	102.66
std	30.03	20.67	21.23	20.63	24.56
Light vehicles					
mean	105.81	105.91	106.57	106.42	105.11
std	28.35	20.46	21.11	20.45	23.08
Heavy vehicles					
mean	90.92	88.47	89.54	89.46	90.38
std	90.92	88.47	89.54	89.46	90.38
σ/vehicles					
mean	20.68	3.22	5.86	4.30	9.79
std	8.96	3.53	3.85	3.24	11.45

**Table 4:** Automatic and manual vehicle counting.

Video Sample	1.1	1.2	1.3	1.4	1.5	2.1	2.2	2.3	2.4	2.5
manual counts	76	72	89	75	85	92	85	102	80	59
automatic detection	72	69	89	70	82	92	83	101	79	58

**Table 5:** Error metrics of the model calculated on the pressure level distribution.

Mean Error	Mean Absolute Error	Mean Absolute Percentage Error	Mean Percentage Error	Root Mean Square Error
−1.0 dBA	3.6 dBA	4.8%	−1.4%	4.4 dBA

**Table 6:** Comparison of mean errors and standard deviations of the errors of the selected models.

Model	Mean Error (On the 10 min *L_eq_*) [dBA]	Standard Deviation of the Errors [dBA]
EAGLE 3.0	−1.7	0.4
Burgess [56]	−1.7	1.8
CSTB [57]	−1.4	0.7
CoRTN [25]	−3.0	0.8
Quartieri et al. [30]	−2.8	0.7
CNOSSOS-EU [55]	−2.4	1.0

## Data Availability

Aggregated data are reported in the paper’s figures and tables. Raw data are available upon reasonable request to the corresponding authors.

## Supplementary Materials

The following supporting information can be downloaded at https://www.mdpi.com/article/10.3390/s25061750/s1, Video S1: A 1 min video sample.
